# Single-Stage Posterior-Only In Situ Fixation and Fusion for Neglected Congenital Vertebral Aplasia With Kyphoscoliosis in a Resource-Limited Setting: A Case Report

**DOI:** 10.7759/cureus.107236

**Published:** 2026-04-17

**Authors:** Prashant Adhikari, Deepak Shrestha, Jeevan K Sharma

**Affiliations:** 1 Orthopaedic Surgery, Hospital for Advanced Medicine and Surgery, Kathmandu, NPL; 2 Orthopaedic Surgery, Asian Institute of Medical Sciences, Faridabad, IND

**Keywords:** congenital kyphoscoliosis, facetectomy, in situ fixation, posterior spinal fusion, resource-limited surgery, vertebral aplasia

## Abstract

Neglected congenital kyphoscoliosis with vertebral aplasia, absent pedicles, and rib anomalies presents a formidable challenge, especially in low-resource settings. We report the case of a 28-year-old man with progressive thoracolumbar deformity, cosmetic concerns, and fatigue. Imaging revealed congenital vertebral aplasia with pedicle agenesis from D10 to D12, a missing left 12th rib, and a dysplastic right L1 pedicle. The patient underwent a single-staged posterior-only surgical correction using basic fluoroscopic guidance. Surgical management included multilevel pedicle screw fixation, wide posterior facetectomies, decompression, foraminotomies, in situ rod contouring, and posterolateral fusion with allograft. No vertebral column resections were performed. Postoperative imaging demonstrated stable correction and alignment. At three months, the patient had significant cosmetic improvement, fatigue resolution, and no neurological deficits. This case demonstrates the utility of posterior-only deformity correction and fusion in complex neglected congenital kyphoscoliosis, using accessible surgical techniques without reliance on advanced intraoperative technology in a resource-limited setting.

## Introduction

Congenital vertebral anomalies represent rare spinal disorders characterized by developmental malformations of the vertebrae. These conditions are described using various overlapping terminologies in the literature, including terms such as segmental spinal dysgenesis, medial spinal aplasia, spinal agenesis, and congenital vertebral dislocation. Despite their rarity, these anomalies are clinically significant due to their potential to cause structural and neurological complications [1]. Congenital kyphoscoliosis associated with vertebral aplasia, pedicle malformations, and rib anomalies is a rare spinal deformity, distinct from complete spinal agenesis. In the absence of timely intervention, this condition typically progresses with advancing age [2]. Multiple classification systems have been proposed to categorize these malformations. Based on these criteria, our case can be identified as thoracolumbar aplasia involving four vertebral levels (D10-L1), accompanied by moderate spinal cord narrowing without associated spinal cord anomalies [3]. Delayed diagnosis often results in the development of a rigid spinal deformity, making surgical management more complex. A posterior-only approach incorporating extensive facetectomies, decompression, in situ rod contouring, and posterolateral fusion can achieve effective correction and stabilization, often eliminating the need for vertebral resection [4]. We report a case of neglected congenital kyphoscoliosis associated with thoracolumbar dysgenesis and a rigid deformity, managed successfully using a single-stage posterior-only surgical approach at the Hospital for Advanced Medicine and Surgery, Kathmandu, Nepal. This case highlights the practicality and efficacy of this method in a resource-limited surgical environment.

## Case presentation

Patient information and history

A 28-year-old man presented with a long-standing thoracolumbar spinal deformity that had progressively worsened. He reported fatigue, postural imbalance, and significant cosmetic concerns. He had delayed treatment due to fear of surgery but denied any neurological complaints.

Clinical findings

**Figure 1:**
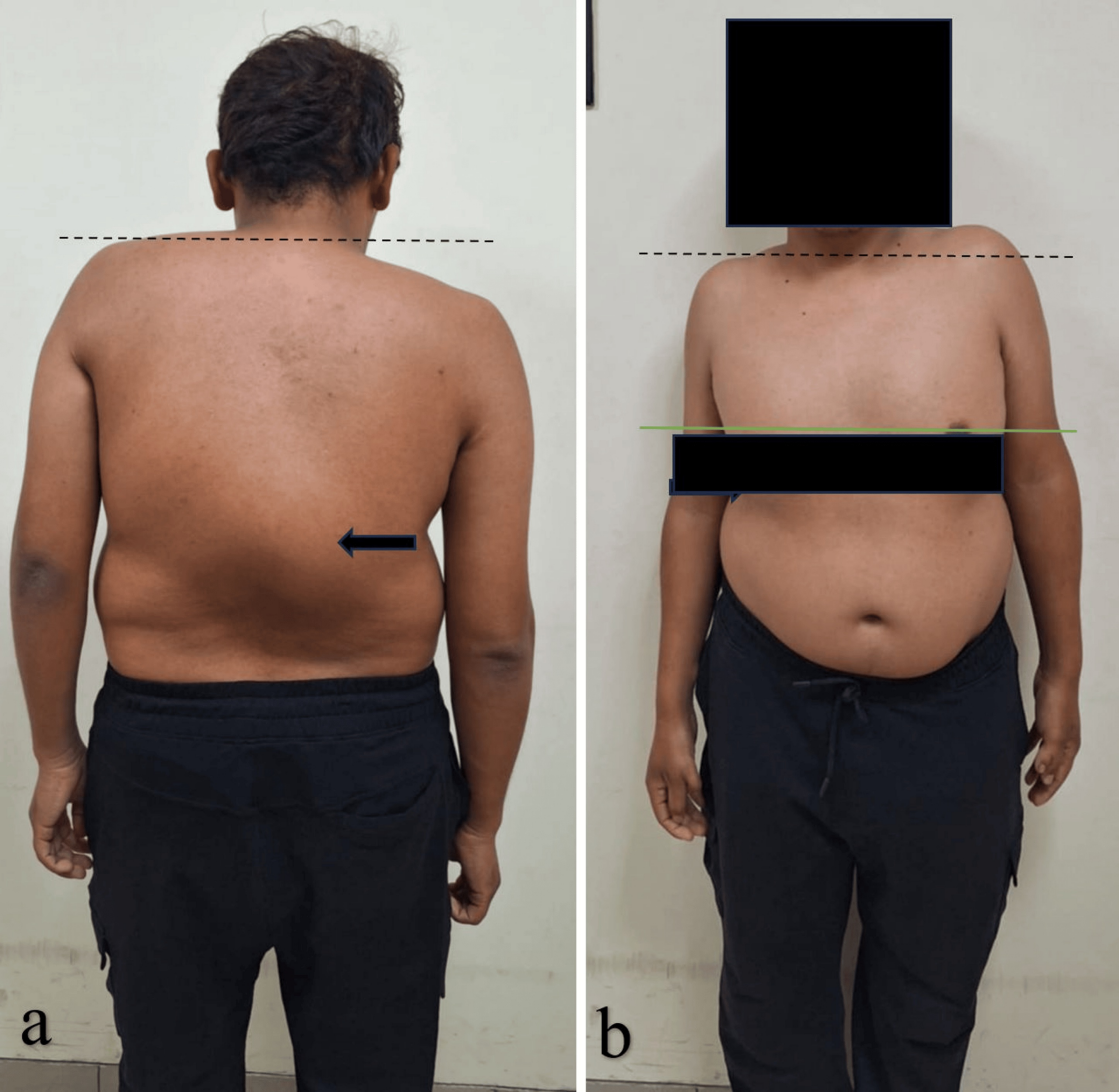
Preoperative clinical photographs of the patient (a) Posterior view showing a prominent kyphotic deformity (black arrow) and noticeable shoulder asymmetry (dotted black line). (b) Anterior view demonstrating marked shoulder height discrepancy (dotted black line), nipple level asymmetry (green line), and evidence of costopelvic impingement (black arrow).

Clinical photographs revealed a prominent right thoracolumbar rib hump with coronal and sagittal imbalance (Figure 1). Neurological examination showed full strength and normal sensory function in all extremities.

Timeline of care

Table 1 presents the clinical timeline of the patient.

**Table 1:** Clinical timeline

Time point	Event
Preoperative imaging	X-ray, MRI, CT, 3D-CT (Figure 2)
Surgery	Single-staged posterior-only correction and fusion
1 month	Postoperative X-ray (Figure 3a)
3 months	Follow-up X-ray and clinical photographs (Figure 3b, Figure 4)

**Figure 2:**
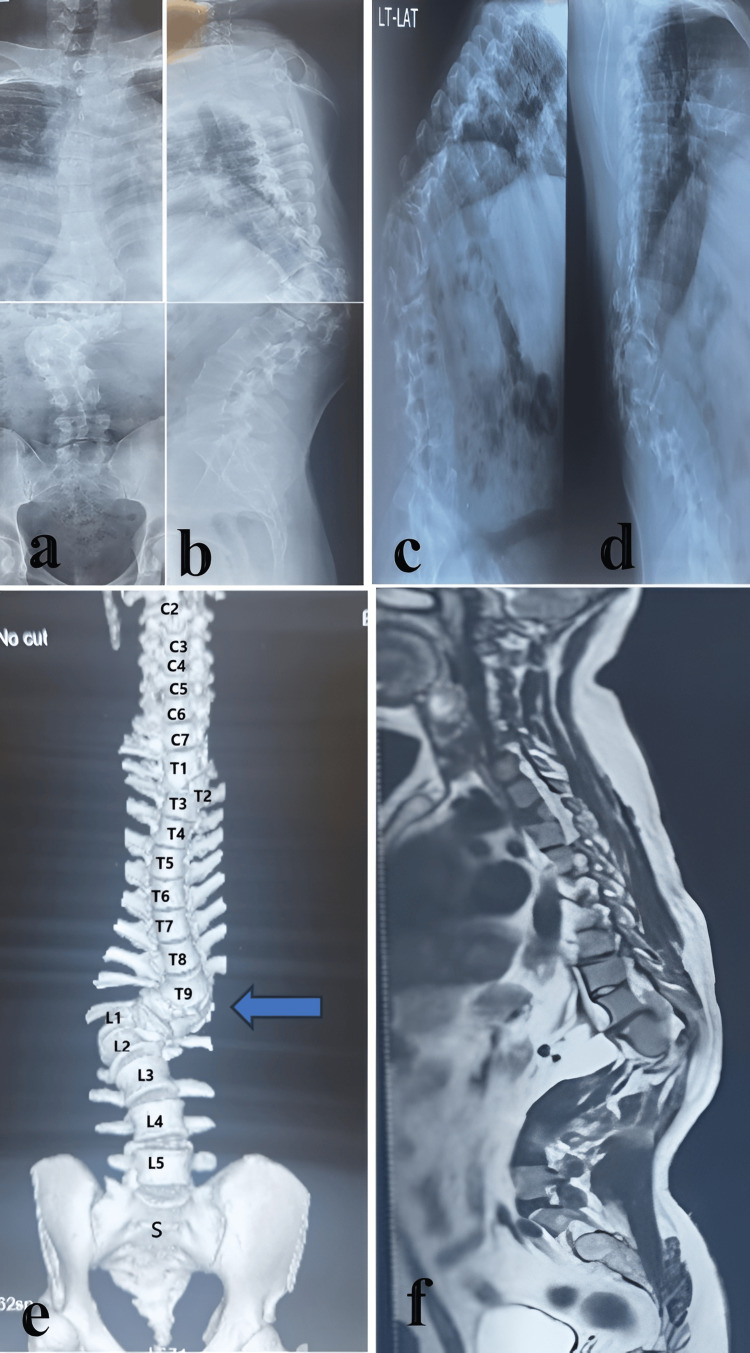
Preoperative imaging of the patient (a and b) Anteroposterior and lateral plain radiographs demonstrating spinal deformity. (c and d) Dynamic flexion and extension X-rays showing rigidity of the deformity. (e) Preoperative 3D-CT reconstruction revealing significant spinal deformity extending from below the T9 level to L1, with vertebral aplasia and absence of the left 12th rib (blue arrow). (f) Preoperative MRI showing no evidence of intraspinal anomalies or spinal cord malformations.

**Figure 3:**
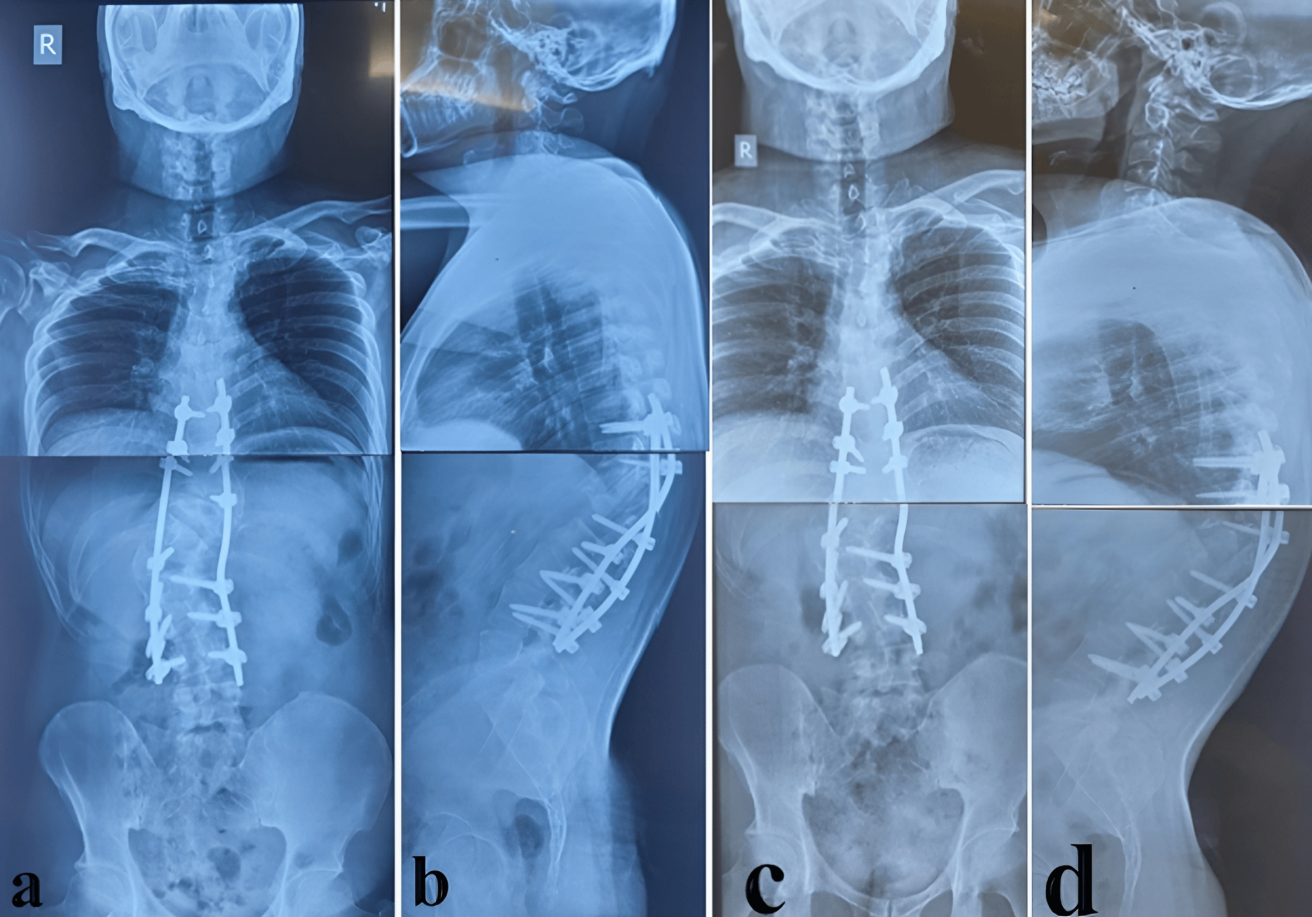
Postoperative radiographs at follow-up (a and b) Anteroposterior and lateral views at one-month follow-up. (c and d) Anteroposterior and lateral views at three-month follow-up demonstrating a significant reduction of the deformity with maintained alignment and no evidence of implant-related complications.

**Figure 4:**
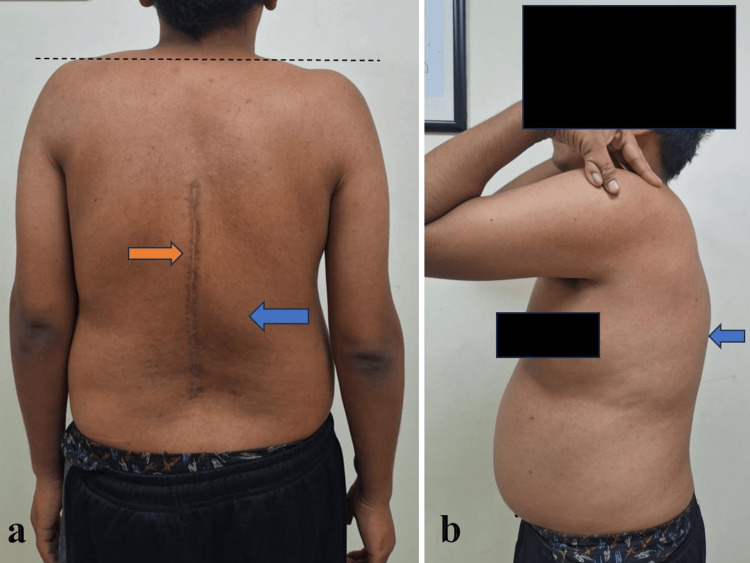
Postoperative clinical photographs (a) Posterior view showing significant reduction in shoulder height discrepancy (dotted black line), a well-healed surgical scar (orange arrow), and decreased kyphotic hump (blue arrow). (b) Left lateral view demonstrating maintained sagittal balance and reduction of kyphosis (blue arrow).

Diagnostic assessment

Radiographs showed severe right thoracolumbar scoliosis with kyphosis and vertebral malalignment with rigid deformity (Figure 2a-2d). CT revealed the absence of D10-D12 pedicles and a dysplastic right L1 pedicle. The left 12th rib was absent (Figure 2e). No intraspinal anomalies were noted on MRI; the spinal cord was displaced toward the concave (left) side (Figure 2f).

Therapeutic challenges

Delayed presentation of congenital deformity with pedicle absence and limited available anchor points for fixation were the challenges encountered. Unilateral rib abnormalities further distorted anatomical landmarks, increasing technical difficulty for safe and effective screw placement.

Therapeutic intervention

Preoperative Planning

Pedicle screws were planned at D7-D9 bilaterally, right L1, and L2-L4 bilaterally. Vertebral resection was avoided. The posterior-only approach with in situ correction and fusion was employed.

Operative Technique

A standard midline posterior approach was performed. Pedicle screws were inserted under fluoroscopic guidance. Wide bilateral facetectomies and soft tissue releases were performed at the apex of deformity to allow for flexibility. Decompression was performed at D10-D12 to protect neural elements due to congenital narrowing. Rods contoured in situ were applied to gradually correct deformity without excessive force. Compression and distraction were applied segmentally for alignment control. Posterolateral fusion using an allograft obtained from posterior elements and facets was completed over decorticated facets and transverse processes. No vertebral resections were undertaken.

Intraoperative Imaging

Standard fluoroscopy was used to verify screw placement and rod alignment. No intraoperative navigation or O‑arm technology was used.

Follow‑up and outcomes

Radiographic

Postoperative X-rays at one and three months showed a stable construct and improved spinal alignment in sagittal (reduction in kyphosis from 85 to 40) and coronal planes (reduction of scoliosis from 80 to 40) (Figure 3).

Clinical

The patient reported improved posture and aesthetics, resolution of fatigue, and no new neurological complaints. He returned to daily activities within three months (Figure 4).

## Discussion

Managing severe, rigid kyphoscoliotic deformities remains a significant challenge for spine surgeons, particularly in settings with limited resources. A variety of surgical strategies and techniques have been documented in the literature to address such complex spinal curves. These range from vertebral column decancellation [5], apical spinal osteotomies [6], and staged procedures utilizing halo-gravity or halo-pelvic traction followed by definitive fixation [7] to more extensive interventions such as vertebral column resections [8].

One of the key physiological concerns with posterior-only fixation in skeletally immature patients is the risk of the crankshaft phenomenon, wherein continued anterior spinal growth can lead to rotational deformity of the instrumented segment [9]. However, in our case, the patient had reached skeletal maturity, which significantly reduced this risk. This, supported by existing evidence in the literature [4,10], justified our decision to proceed with a posterior-only approach for deformity correction.

This case illustrates the safe and effective correction of a neglected complex congenital spinal deformity using a posterior-only approach. The presence of vertebral aplasia, pedicle agenesis, and rib anomalies added complexity to surgical planning. However, with meticulous dissection, adequate decompression, wide posterior releases, and in situ rod application, significant correction was achieved without the need for vertebral column resection or advanced intraoperative imaging.

Facetectomy-based flexibility, combined with segmental compression and controlled rod contouring, proved sufficient for correcting both coronal and sagittal components of the deformity [4]. This technique offers a reproducible solution for surgeons operating in environments where equipment such as O-arm, navigation, or neuromonitoring may not be available. Limitations include the short duration of follow-up and the inherent challenges of generalizing a single-case outcome in our setting. Nonetheless, this approach minimizes surgical risk, preserves neural elements, and avoids the morbidity associated with more aggressive osteotomies or resections.

## Conclusions

This case highlights that a single-stage posterior-only approach can be an effective and practical strategy for managing neglected congenital kyphoscoliosis associated with vertebral aplasia and pedicle agenesis, even in resource-limited settings. Adequate deformity correction and spinal stabilization were achieved through meticulous surgical planning, wide posterior releases, decompression, and in situ rod contouring, without the need for complex osteotomies, vertebral column resection, or advanced intraoperative technologies.

The favorable clinical and radiological outcomes in this patient demonstrate that, in skeletally mature individuals, posterior-only fixation and fusion can provide satisfactory correction while minimizing surgical morbidity and preserving neurological function. This approach offers a reproducible and accessible solution for spine surgeons working in constrained environments. However, longer follow-up and larger case series are necessary to validate the durability and broader applicability of this technique.
